# Job satisfaction and psychological factors influence the caring behavior in nurses in Sierra Leone: a cross-sectional study

**DOI:** 10.3389/fpsyg.2024.1418260

**Published:** 2024-09-05

**Authors:** Xiao Wang, Juan Xue, Aidi Zhang, Yaoyue Luo, Ouying Chen, Caixia Liu, Jie Zhang, Meirong Wu

**Affiliations:** ^1^Nursing Department, The Third Xiangya Hospital, Central South University, Changsha, Hunan, China; ^2^School of Nursing, Hunan University of Chinese Medicine, Changsha, Hunan, China

**Keywords:** caring behaviors, resilience, anxiety, job satisfaction, nursing quality

## Abstract

**Objective:**

Identifying the status of caring behavior and its influencing factors in nurses is crucial for improving the quality of care for patients. However, there is a lack of studies on this in Sierra Leone. This study explored the status of caring behavior and associated factors in nurses working in Sierra Leone.

**Study design:**

A cross-sectional descriptive survey was conducted from October 3 to December 15, 2022, with clinical nurses recruited through convenience sampling.

**Methods:**

The participants included 360 nurses from various nursing departments from 12 hospitals in Sierra Leone. Measurements included a general information questionnaire, the Caring Behavior Inventory, Connor-Davidson Resilience Scale, McCloskey/Mueller Satisfaction Scale and Zung’s Self-Rating Anxiety Scale. Descriptive statistics, one-way analysis of variance and independent-sample *t*-tests, Pearson’s correlation analyses, and multiple regression analyses were performed.

**Results:**

Caring behavior score was 128.97 (SD 11.967); it significantly and positively correlated with job satisfaction and resilience and negatively correlated with anxiety. Linear regression analysis showed that resilience, job satisfaction, anxiety, daily working time, and position were the main predictors of caring behavior.

**Conclusion:**

The level of caring behavior in nurses in Sierra Leone was moderate. Resilience and job satisfaction significantly and positively predicted nurses’ caring behavior. Anxiety, daily working time, and position are important factors of caring behaviors.

**Implications for practice:**

It is suggested to create a conducive working environment, reduce the excessive workload of nurses, enhance their positive psychological state, and their job satisfaction by providing recognition and rewards to improve the level of nurses’ caring behavior.

## 1 Introduction

The Republic of Sierra Leone is located on the west coast of Africa and has a poor health system owing to a long civil war (1991–2002) and the Ebola epidemic (2014–2015). The health system in Sierra Leone is fragile and plagued by inadequate human resources, together with a history of low and irregular remuneration for healthcare professionals (Wurie et al., 2016). The life expectancy at birth in Sierra Leone was 52.2 years in 2019, the lowest globally (Programme, 2019), while infant mortality was the second worst, better than that in only the Central African Republic, and under-5 mortality was better than that in only three other countries (Programme, 2019). Currently, the health indicators in Sierra Leone are the worst worldwide. Nurse density in 2016 was also one of the lowest worldwide, with just 0.2 nurses and midwives per 1,000 people (Bank, 2023). Under such conditions, nurses play an important role in the medical treatment of patients.

According to Watson and Jean, caring is intertwined with the meaning of nursing in that it cannot be separated from nursing (Watson, 1979). Caring behavior is the foundation of nursing. Since nurses spend a lot of time interacting with patients and their families, their caring behavior is important when providing nursing treatment and psychological comfort and conducting other interventions. Research indicates that nurses’ caring behavior influences care quality, patient satisfaction, and readmission, all of which are important indicators of service quality (Hajinezhad et al., 2008). Due to poor health conditions and the lack of nurses in Sierra Leone, nurses’ caring behavior is particularly important for patients and requires urgent attention. However, there are no relevant studies exploring nurses’ caring behavior in Sierra Leone, much less on proper measures to promote caring behavior in nurses. Therefore, identifying the level of caring behavior in nurses and its influencing factors is crucial for improving the quality of care for patients.

Some scholars have discussed the caring behavior of nurses in different countries and regions (Papastavrou et al., 2012; Shen et al., 2020); these results differ due to varying national conditions. A study conducted in Eastern Ethiopia showed that 63.4% of participants had a good perception of caring behavior (Fikre et al., 2022). According to results from the Jimma University specialized hospital, 82.9% of nurses ranked the technical-professional aspects of caring behavior as the most important (Oluma and Abadiga, 2020). These studies highlight a common finding that a high level of caring behavior in nurses implies treating patients with respect and knowing nursing operations and equipment handling. This shows that identifying the influencing factors of nurses’ caring behavior is pivotal for shaping good nursing care, which remains an important issue for the international nursing community. Researches (Kibret et al., 2022; Oluma and Abadiga, 2020; Taylan et al., 2021) showed that caring behavior in nurses may be influenced by factors including sociodemographic characteristics, workload, educational background, job satisfaction, staffing issues, interaction with co-workers/doctors, working environment, shift work, and mental health. However, no similar studies have been conducted in Sierra Leone.

## 2 Theoretical justification

The study was guided by the Neuman Systems Model, the model diagram breaks down the meta-paradigm of nursing into the four concepts of environment, person, nursing, health. Within the Neuman systems model, the individual is viewed as a client or client system composed of innate features embedded within a specific structure. The client system develops a series of defenses that are used as protection to interact with the environment called “lines of defense.” The normal line of defense can be interpreted as the client’s usual level of wellness (Hannoodee and Dhamoon, 2023). The Neuman Systems Model indicated that environmental stress and reactions to stress are closely associated with the wellness of the client system (Shen et al., 2020). In this study, we considered nurses working in Sierra Leone as the client system and caring behavior as the core of nursing. Stressors can diminish nurses’ caring behavior, and personal states (e.g., mental health), and occupational characteristics (e.g., job satisfaction and workload) can be considered stressors associated with nurses’ caring behavior. Regarding mental health, resilience and anxiety were considered as positive and negative mental states, respectively.

Job satisfaction and caring behaviors, psychological factors (resilience, anxiety) and caring behaviors, and the relationship among them, are of critical importance as they are linked to nurses’ quality of life as professionals, quality of care provided, and patients’ well-being. Researchers have given less attention to the caring behaviors in nursing, especially nurses in Sierra Leone. This in turn creates patient dissatisfaction and poor nursing care delivery, resulting in a poor prognosis. Hence, guided by the Neuman Systems Model, it is essential to better understand the status of caring behavior in nurses working in Sierra Leone, to explore the effects of occupational characteristics, job satisfaction, resilience, and anxiety on caring behaviors among nurses in the hope of providing suggestions to promote nurses’ professional well-being; and to enhance the quality of nurses’ caring behaviors for patients in Sierra Leone, as well as to recommend better professional care behavior in the study setting.

## 3 Methods

### 3.1 Aim

This research aimed to explore the level of caring behavior in nurses working in Sierra Leone and to examine the influence of demographic characteristics, resilience, anxiety, and job satisfaction on caring behavior.

### 3.2 Design

A cross-sectional descriptive survey was conducted in accordance with the Strengthening the Reporting of Observational Studies in Epidemiology (STROBE) guidelines (von Elm et al., 2007) (see Supplementary file S1).

### 3.3 Participants

Clinical nurses from various departments in 12 hospitals in Sierra Leone were recruited using convenience sampling. The inclusion criteria were as follows: (1) currently engaged in clinical practice and (2) willing to participate in the study. Nurses who had participated in other relevant studies were excluded.

A fixed model in G power 3.1 (Faul et al., 2009) was used to calculate the sample size for linear multiple regression. The final sample size was 222 with an effect size α = 0.05, *f*^2^ = 0.15, 1−β = 0.95, and 20 explanatory variables (17 demographic variables and resilience, job satisfaction, and anxiety). Considering 10% loss at follow-up and owing to sampling error, the sample size was expanded to 244.

### 3.4 Data collection

#### 3.4.1 Sociodemographic characteristics

A questionnaire was self-compiled to collect participants’ demographic characteristics data. It included sex, age, marital status, education level, type of hospital, title, position, department, years of nursing, nursing shift scheduling pattern, daily working time, weekly working time, childbirth, monthly income, and sleep duration.

#### 3.4.2 Caring behaviors inventory-24

The Caring Behaviors Inventory (CBI) was developed by Wolf (1986) and revised by Wu (CBI-24) (Wu et al., 2006). The CBI-24 contains 24 items in four subscales (assurance of human presence, knowledge and skill, respectful deference to others, and positive connectedness). A 6-point Likert scale is used (1 = never to 6 = always), and higher scores indicate more specific caring behavior expressed by nurses. Following previous studies (Fikre et al., 2022), good and poor caring behaviors were defined by a score equal to or higher than and lower than the mean score, respectively. The scale has good reliability and validity. Cronbach’s alpha for the scale in this study was 0.896.

#### 3.4.3 Connor-Davidson resilience scale

The Connor-Davidson Resilience Scale (CD-RISC) (Connor and Davidson, 2003) was used to measure nurses’ resilience. The CD-RISC comprises 25 items in three dimensions (tenacity, strength, and optimism). A 5-point Likert scale (0 = never to 4 = almost always) is used, and higher scores indicate a higher level of resilience. The scale has a good reliability and validity (Connor and Davidson, 2003). Cronbach’s alpha in this study was 0.953.

#### 3.4.4 McCloskey/Mueller satisfaction scale

The McCloskey/Mueller Satisfaction Scale (MMSS) was developed by Mueller and Mccloskey (1990) to measure nurses’ job satisfaction. The scale has 25 items and five subscales (work culture and conditions, scheduling and family/work balance, collateral relationships, extrinsic rewards, and professional opportunities) using a 5-point Likert scale (1 = very dissatisfied to 5 = very satisfied). The MMSS has shown good reliability and validity in several countries (Gurková et al., 2013). Cronbach’s alpha in this study was 0.943.

#### 3.4.5 Self-rating anxiety scale

The Self-Rating Anxiety Scale (SAS) developed by Zung was used to measure participants’ anxiety. It is a widely used standardized screening test that can easily measure anxiety disorders in healthy individuals (Byeon, 2021) using 20 items rated on a 4-point Likert scale (1 = none or a little of the time to 4 = most or all the time). A higher score indicates a higher level of anxiety. Summation of the item scores was multiplied by 1.25 to obtain the standard score. A score ≥ 50 was used as the threshold for determining anxiety. Cronbach’s alpha in this study was 0.649.

### 3.5 Procedures

The data were collected from October 3 to December 15, 2022, when the author Xiao Wang was a member of a Chinese medical team assisting in Sierra Leone. First, Xiao Wang informed the head nurses in the China-Sierra Leone Friendship Hospital of the purpose of the study and obtained their permission to recruit nurses. Thereafter, Xiao Wang introduced the questionnaires to a research nurse working at the hospital and explained how to complete them. Finally, Xiao Wang distributed a link to access the questionnaires on Qualtrics, a widely used electronic questionnaire survey tool, to the research nurse. Eligible participants contacted Xiao Wang and the research nurse to participate in the study.

### 3.6 Ethical considerations

This study was approved by the Institutional Review Board (IRB) of the Xiangya School of Nursing, Central South University in March 2020 before data collection (No: E202027). Prior to responding to the survey, participants were informed of the purpose and process of the study as well as of their rights and obligations to participate and signed an online informed consent.

### 3.7 Data analysis

IBM SPSS Statistics (version 20.0; IBM, Chicago, IL, United States) was used for data analyses. We used means, standard deviations, frequencies, and percentages to analyze demographic information, caring behavior, job satisfaction, resilience, and anxiety. Pearson’s correlation analyses were used for correlations between two variables, and *t*-tests and analysis of variance (ANOVA) were used to analyze the influence of sociodemographic characteristic on caring behaviors. Multiple linear regression analysis was used to identify the influence of sociodemographic variables, resilience, job satisfaction, and anxiety on caring behavior. All statistical tests were two-sided (α = 0.05).

## 4 Results

### 4.1 Sample characteristics

A total of 384 nurses participated in this survey. Of them, partially-missing data were reported by 24 nurses. Therefore, 360 nurses’ data were included in the analysis (valid response rate: 93.8%). Of them, 80.8% were women, majority were between 26 and 35 years of age, and 90.3% worked in public hospitals. Other sociodemographic characteristics are presented in Table 1.

**Table 1:** Socio-demographic characteristics of participants and differences among variables.

Variables	Categories	*N*	Mean	SD	*t/F*	*p*
**Sex**					−1.089	0.277
	Male	69	127.57	15.013		
	Female	291	129.31	11.128		
**Age**						
	18–25	68	131.87	10.886	2.051	0.106
	26–35	178	128.80	12.121		
	36–45	99	127.24	12.380		
	≥46	15	129.33	10.588		
**Marital status**						
	Married	183	128.57	13.527	1.821	0.143
	Member of an unmarried couple	10	123.00	5.354		
	Divorced or separated	9	135.22	6.648		
	Single	158	129.47	10.320		
**Education level**					6.585	0.000*
	Undergraduate	230	127.80			
	Graduated but still work on degree	73	134.44			
	Associate degree or less	12	126.75			
	Bachelor degree	35	128.91			
	Master degree or above	10	119.00			
**Type of hospital**					−0.340	0.734
	Public hospitals	325	128.90	12.053		
	Private hospital	35	129.63	11.275		
**Title**					2.020	0.091
	Newly qualified nurse	93	129.96	10.569		
	Experienced nurse	188	129.84	11.661		
	Clinical nurse specialist/nurse practitioner	35	126.14	12.717		
	Nurse consultant	2	119.00	7.071		
	Others (specify)	42	125.76	14.826		
**Position**					2.425	0.035*
	Staff nurse	197	130.20	11.232		
	Sister	29	130.69	10.761		
	Ward manager	32	130.78	11.631		
	Matron/lead nurse	13	126.92	12.770		
	Deputy nursing director/nursing director	4	125.25	32.212		
	Others (specify)	85	125.36	12.187		
**Department**					1.987	0.056
	Emergency department	52	131.12	12.513		
	Pediatrics department	62	125.74	12.786		
	Surgical department	52	131.12	10.165		
	Medical department	68	129.09	10.598		
	Gynecology and obstetric department	49	130.65	10.990		
	Intensive care unit	13	132.62	7.124		
	Operation theater	24	124.71	14.574		
	Others (specify)	40	127.55	13.786		
**Years of nursing**					1.562	0.198
	≤1 year	98	128.74	12.868		
	2–5 years	127	129.90	12.078		
	6–10 years	81	129.83	11.182		
	>10 years	54	125.94	10.896		
**Nursing shift scheduling pattern**					0.686	0.561
	I work day shifts only	99	129.87	14.164		
	1–2 night shift	148	129.12	11.586		
	3–4 night shift	90	128.49	10.012		
	>4 (specify)	23	126.09	11.281		
**Daily working time**					4.360	0.013*
	≤8 h	197	130.61	12.197		
	8–12 h	154	127.14	11.540		
	>12 h	9	124.56	8.988		
**Weekly working time**					3.033	0.018*
	≤35 h	93	132.46	12.259		
	36–40 h	132	127.88	12.524		
	41–45 h	39	126.85	10.701		
	46–50 h	80	128.50	11.060		
	>50 h (specify)	16	125.31	9.379		
**Childbirth**					0.837	0.502
	I’m still work on it	82	129.73	11.076		
	1–2 kids	191	129.09	11.972		
	3–4 kids	63	127.79	13.303		
	>5 kids	14	131.71	10.321		
	Others (specify)	10	124.10	12.476		
**Monthly income**					2.723	0.013*
	≤500 Nle	108	129.24	12.838		
	501–2,000 Nle	87	129.67	10.571		
	2,001–3,500 Nle	73	130.58	11.589		
	3,501–5,000 Nle	30	131.90	10.816		
	5,001–6,500 Nle	4	115.25	27.789		
	>6,500 Nle	8	122.63	5.755		
	Others (specify)	50	125.22	11.320		
**Sleep duration**					4.596	0.011*
	≤6 h	132	131.37	12.432		
	6–8 h	210	127.40	11.733		
	8–10 h	18	129.78	8.048		

*p < 0.05.

### 4.2 Caring behavior and its association with job satisfaction, resilience, and anxiety

The mean caring behavior score was 128.97 (SD 11.967). The data revealed that 58.9% of nurses had good caring behavior. The average scores for job satisfaction, resilience, and anxiety were 80.57 (SD 16.713), 73.08 (SD 18.754), and 47.77 (SD 8.178), respectively. Table 2 presents the detailed results.

**Table 2:** Mean and standard deviations of variables.

Variables	Mean	SD
Caring behaviors	128.97	11.967
Assurance	42.86	4.956
Knowledge and skill	27.79	2.596
Respect	32.64	3.262
Connectedness	25.69	3.789
Job satisfactory	80.57	16.713
Work culture and conditions	24.88	4.840
Scheduling and family/work balance	26.20	5.844
Extrinsic rewards	7.70	3.131
Collegial relationships	16.60	3.750
Professional opportunities	5.19	2.327
Resilience	73.08	18.754
Tenacity	37.59	10.171
Strength	23.96	6.446
Optimism	11.52	3.186
Anxiety	47.77	8.178

Regarding the association between the variables, caring behavior positively correlated with job satisfaction (*r* = 0.238, *p* < 0.001) and resilience (*r* = 0.246, *p* < 0.001) and negatively associated with anxiety (*r* = −0.161, *p* < 0.001) (Table 3).

**Table 3:** Pearson’s correlations (*p*-values) between variables among nurses.

Variables	Caring behaviors	Resilience	Anxiety	Job satisfactory
Caring behaviors	1			
Resilience	0.246*	1		
Anxiety	−0.161*	−0.103	1	
Job satisfactory	0.238*	0.031	−0.077	1

*p < 0.05.

### 4.3 Univariate analyses of the factors associated with caring behavior

ANOVA and *t*-tests indicated that education level, position, daily working time, weekly working time, monthly income, and sleep duration influenced nurses’ caring behavior (Table 1).

### 4.4 Regression analysis

Several sociodemographic variables, job satisfaction, resilience, and anxiety were associated with caring behavior. Regression analysis was conducted with caring behavior as the outcome variable, and job satisfaction, resilience, anxiety, and other sociodemographic factors that reported significant differences as explanatory variables. Subsequently, the variables were screened using the stepwise method, and regression analysis was performed (entered α = 0.10, exited α = 0.15). The results indicated that high levels of job satisfaction and resilience, low levels of anxiety, low job positions, and shorter working hours were the main predictors of a high level of caring behavior (*F* = 13.959, *p* = 0.000, *R*^2^ = 0.165, adjusted *R*^2^ = 0.153) (Table 4).

**Table 4:** Multiple linear regression analysis examining covariates of caring behaviors.

Variables	Nonstandardized β coefficient		Standardized β coefficient		
	β	SE		*t*	*p*
Consent	122.300	5.685		21.513	0.000*
Resilience	0.136	0.031	0.214	4.366	0.000*
Job satisfactory	0.144	0.035	0.201	4.106	0.000*
Post	−0.844	0.282	−0.147	−2.994	0.003*
Working time of day	−2.797	1.065	−0.128	−2.627	0.009*
Anxiety	−0.180	0.072	−0.123	−2.504	0.013*

*p < 0.05.

## 5 Discussion

This study examined the level of caring behavior and its influencing factors in nurses working in Sierra Leone. The results indicated that 58.9% of the nurses had a good level of caring behavior which was predicted by high levels of job satisfaction and resilience, low levels of anxiety, low position, and shorter working hours. The findings provide an important basis for improving nursing care behavior in Sierra Leone.

The percentage of nurses with a good level of caring behavior (58.9%) was lower than that in a study conducted in Harar City Hospitals in Eastern Ethiopia (63.4%) (Fikre et al., 2022,). These may be due to the differences in medical conditions, study methodologies, nature of the organization, and sample size. When the average scores of each subscale were arranged in a descending order, positive connectedness ranked the lowest, consistent with the findings of a previous study (Omari et al., 2013), below knowledge and skill, and consistent with the findings of previous studies (Labrague et al., 2017; Sarafis et al., 2016; Shen et al., 2020), assurance of human presence ranked the highest. Positive connectedness implies that the essence of nursing is to meet the basic needs of patients, which confirms Watson’s conceptions that nursing actions are intended to meet individuals’ basic needs and that it is the best interpretation of caring (Watson, 2002). This also reflects the commitment of nurses to nursing care in Sierra Leone, despite the lack of adequate medical conditions and a serious shortage of nurses. Therefore, low positive connectedness could be related to nurses’ lack of time and overwork, resulting in an imbalance of patient flow and human resources. It is recommended that the Government of Sierra Leone train nurses to improve this situation.

The present study revealed that job satisfaction is an important predictor of nurses’ caring behavior. Previous studies have also indicated that job satisfaction is associated with and influences caring behavior in nurses, especially in critical care units and surgical wards (Amendolair, 2012; De Los and Labrague, 2021; Plevová et al., 2021). When nurses are satisfied with their work, their confidence in their ability to provide high-quality care to patients is strengthened. Meanwhile, several studies have shown that workplace and personal satisfaction have a major impact on positive work behavior (Akinwale and George, 2020). Therefore, job satisfaction can motivate nurses to improve their caring behavior. Organizations must develop strategies that support and value a caring environment so that nurses enjoy their work, improve job satisfaction, and increase loyalty and commitment to their work.

This study also found that high resilience and low anxiety correlated with good levels of caring behavior. As an individual’s internal motivation to mobilize other resources in the face of setbacks, resilience is a key factor in helping people cope with stress and develop effective coping skills (Zhang et al., 2022). Nurses with high levels of resilience usually remain positive and regard the difficulties encountered at work as a part of their lives, which help them cope with hardships successfully. Consequently, they treat patients with a positive attitude, meet their needs during the nursing process, and demonstrate a high level of caring behavior. A previous study also showed that a high level of resilience can help nurses reduce the emotional exhaustion resulting from providing patient care and increase job satisfaction (Yu et al., 2019). Therefore, effective strategies should be implemented to improve resilience among nurses. In contrast, anxiety is a manifestation of nurses’ negative mental states.

A previous study also indicated that a high level of anxiety was associated with poor caring behavior (Sarafis et al., 2016). A negative mental state affects nurses’ work attitudes and enthusiasm and unconsciously penetrates negative emotions in the process of nursing, seriously affecting their caring behavior. Nursing managers should pay close attention to the mental health of nurses, promote positive mental states, and avoid negative mental states.

This study showed that the longer the nurses’ daily working hours, the worse their caring behavior. This is consistent with the results obtained in Eastern Ethiopia (Fikre et al., 2022). This implies that workload influences nurses’ caring behavior. Although nurses in Sierra Leone cherish their jobs, excessive workloads can lead to burnout, which is negatively associated with their professional efficacy, resulting in poor caring behavior. However, research has shown that workload and caring behavior are not correlated (Rizkianti and Haryani, 2020). This may be attributed to the differences in nurses’ salaries and organizational environments. This study also revealed that nurses in higher positions exhibited poorer caring behaviors. In Sierra Leone, nurses in higher positions may have less access to patients which causes lower levels of caring behavior. Therefore, the workload of nurses in lower positions should be considered.

## 6 Recommendation

Considering the poor medical conditions and shortage of nurses in Sierra Leone, the findings can help improve nurses’ caring behaviors. Nursing administrators should implement strategies such as create conducive working environments, reduce the excessive workload of nurses, especially nurses in lower positions; Second, hospital and nursing administrators can conduct several mental health education programs on resilience, positive coping skills, and combating negative emotions to enhance positive mental health. Third, some measures can be implemented to improve nurses’ job satisfaction at hospital level. Overall, the study focused on caring behavior in nurses working in Sierra Leone to help improve nursing quality for African patients and promoting their rehabilitation with limited health resources. In the future, we can construct relevant intervention measures according to the factors affecting caring behavior and improve nurses’ caring behavior.

## 7 Limitations

This study had several limitations. First, the nurses were recruited from hospitals in Sierra Leone through convenience sampling, which limited the generalizability of the findings. Future studies should consider expanding the geographic scope of the sample. Second, it only analyzed some influencing factors of caring behavior. Further studies should consider more factors, such as relationships between colleagues, organizational environment, and working patterns.

## 8 Conclusion

In this study, the level of caring behavior in nurses in Sierra Leone was moderate. Resilience and job satisfaction significantly and positively predicted nurses’ caring behavior. Anxiety, daily working time, and position are important factors influencing caring behaviors. Nursing administrators should realize the value of resilience and job satisfaction and improve them to promote caring behavior in nurses. Moreover, workload of nurses in lower positions requires urgent attention. This study also provides a basis for future research on caring behavior of nurses in Sierra Leone.

## Data Availability

The raw data supporting the conclusions of this article will be made available by the authors, without undue reservation.
